# Carcinome colloïde du sein: à propos d'un cas

**DOI:** 10.11604/pamj.2013.16.93.3444

**Published:** 2013-11-12

**Authors:** Kamilia Laabadi, Sofia Jayi, Fatimazohra Fdili Alaoui, Hakima Bouguern, Hikmat Chaara, My Abdelilah Melhouf

**Affiliations:** 1 Service de gynéco, obstétrique 2, CHU HASSAN II, Fes, Maroc

**Keywords:** Cancer du sein chez l'homme, carcinome colloïde, immunohistochimie, radiothérapie, breast cancer in man, colloid carcinoma, immunohistochemistry, radiotherapy

## Abstract

Nous rapportons le cas d'une tumeur colloïde du sein chez un homme. Cette situation rare interpelle par son mode de découverte. Nous avons pris en charge un homme de 60 ans atteint d'une lésion rétro-aréolaire droite classée cliniquement T4b N1 M0 et suspecte radiologiquement. L'analyse histologique (microbiopsie) a conclu à un carcinome colloïde muqueux associé à une petite composante canalaire classique de grade I de SBR du sein. Les traitements complémentaires associent mastectomie, curage, chimiothérapie, radiothérapie et hormonothérapie. Le cancer du sein est rare chez l'homme. Le carcinome colloïde est exceptionnel puisqu'il représente seulement 1 à 6% de l'ensemble des cancers du sein. Il est encore plus rare chez l'homme. Ces tumeurs touchent une population spécifique et ont un meilleur pronostic que les autres types prépondérant dans les cancers du sein chez l'homme. A travers cette observation et une revue de la littérature, nous essaierons de discuter les principales caractéristiques anatomo-cliniques et évolutives de cette forme rare du cancer du sein.

## Introduction

Le carcinome colloïde du sein, appelé aussi mucineux ou gélatineux, est une forme histologique rare de cancer qui représente 1 à 6% de l'ensemble des cancers du sein [1]. Il est caractérisé par la production de mucus extracellulaire [1]. Histologiquement. On distingue 2 types de carcinome colloïde: le carcinome colloïde pur dans lequel on ne trouve pas de composante de carcinome canalaire infiltrant et le carcinome colloïde mixte qui associe des foyers de carcinome canalaire infiltrant à côté de la composante colloïde [1], cette distinction est capitale du fait de sa valeur pronostique. Le profil évolutif du carcinome colloïde du sein est très variable selon qu'il s'agit d'une forme pure ou mixte [1]. A travers une observation rare de carcinome colloïde survenant chez un homme, notre objectif est de préciser les particularités anatomiques, immunohistochimiques et évolutives du carcinome colloïde du sein.

## Patient et observation

**Figure 1:**
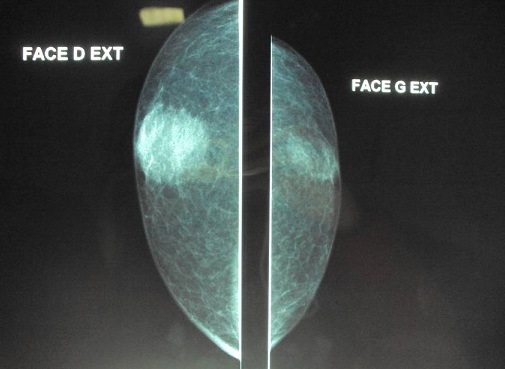
Mammographie de face opacité rétroaréolaire droite de forme arrondie bien limitée de contours irréguliers, flous, avec fines spicules, hétérogènes sans foyer de microcalcifications. Cette opacité est classée ACR 4

**Figure 2:**
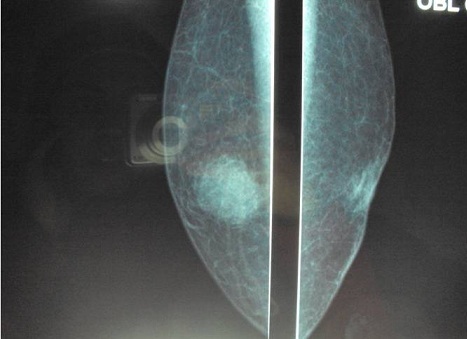
Cliché mammographique de profil montrant les mémes caractéristique de l'opacité sus décrite

Patient de 60 ans, sans ATCDs pathologiques notables, admis pour prise en charge d'un nodule du sein droit évoluant depuis 2 ans et chez qui l'examen clinique objective un nodule de 2/2 cm, rétroareolaire droit classé cliniquement T4bN1MO. La mammographie trouve une opacité rétroaréolaire droite de forme arrondie bien limitée de contours irréguliers, flous, avec fines spicules, hétérogènes sans foyer de microcalcifications. Cette opacité est classée ACR 4 (Figure 1, Figure 2). Le complément échographique objective une lésion rétro-aréolaire droite mal limitée de contours spiculés, hypoéchogène hétérogène mesurant 20mm de grand axe associé à des adénopathies axillaires droites suspectes également.

**Figure 3:**
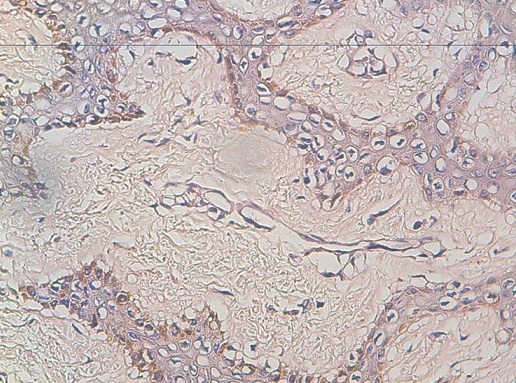
Aspect histologique du carcinome colloide

La microbiopsie du nodule est en faveur d'un carcinome colloïde muqueux associé à une petite composante canalaire classique de grade I de SBR (2MSBR, pas vu d'emboles vasculaires). Un bilan d'extension fait d'une radiographie thoracique et d'une échographie hépatique, est sans particularité. Un haschteid modifié a été réalisé, dont le résultat histologique est en faveur d'un carcinome mucineux avec une composante de carcinome canalaire infiltrant, de grade I de SBR, 2MSBR, mesurant 3,4 cm à noter l'absence de carcinome in situ (Figure 3). Le curage ganglionnaire a ramené 37 ganglions dont 28 sont métastatiques avec emboles vasculaires et rupture capsulaire. La tumeur est classée p T2N3Mo. L'étude des récepteurs ‘strogènique montre un marquage de 100%, l'étude des récepteurs progestéroniques montre un marquage de 90% alors que l'HER2 est négatif et le Ki 67 est à 30%. Une chimiothérapie a été démarrée et on prévoit une radiothérapie et une hormonothérapie.

## Discussion

Le cancer du sein chez l'homme est une maladie rare. Sa prévalence est d'un pour 100000 individus et il représente moins de 1% des cancers du sein et moins de 1% de l'ensemble des néoplasies masculines [2]. L'incidence de cette pathologie semble relativement stable en Europe [3], mais Giordano et al montrent une augmentation de 26% entre 1973 et 1998 aux Etats Unis [4]. L'âge moyen lors du diagnostic varie entre 63 et 71 ans [5] ce qui correspond à cinq ans de plus par rapport à l'âge de découverte du cancer du sein chez la femme avec des extrêmes allant de 5 à 93 ans [3, 4]. Sa distribution est unimodale. Dans notre série l'âge dépasse 50 ans avec des extrêmes de 56 et 79 ans.

La plus grande série publiée de sujets masculins compile dix cas sur une période de 29 ans auxquels on peut ajouter les cas de Jundt et Papotti [1]. Le carcinome colloïde est une variante histologique particulière de carcinome mammaire, décrite la première fois en 1826 par Geschickter [1]. La forme pure représente 0,8 à 1,5% de l'ensemble des carcinomes invasifs du sein [1] et 33 à 95% de l'ensemble des carcinomes colloïdes du sein [1]. Ce faible taux serait dû à une méconnaissance du type mixte et à son inclusion probable dans le type canalaire infiltrant [6]. La forme pure du carcinome colloïde survient à un âge moyen allant de 49 à 67 ans [7]. Elle est plus tardive que la forme mixte [1].

Le mode de révélation le plus fréquent est un nodule mammaire palpable (plus de 80% des cas), comme c'est le cas de notre patient [2, 4, 8], habituellement dans le quadrant supéro-externe [6, 8–10]. La bilatéralité est rarement décrite dans la littérature [8, 11] alors que certains cas de carcinome multifocal ont été rapportés dans la littérature [8]. La taille moyenne tumorale rapportée est de 1,5 cm avec des extrêmes allant de 0,3 à 19 cm [1, 7], la taille tumorale dans notre cas est de 2 cm. La majorité des tumeurs (96%) étaient de stade T1 ou T2 selon la classification TNM [7]. Les adénopathies palpables sont plus fréquentes dans le carcinome colloïde mixte PAR% dans la forme pure [1]. Notre patient a consulté pour une tumeur classée T4b N1.

L'aspect mammographique le plus évocateur est celui d'une opacité dense, circonscrite ou polylobé, à contours finement irréguliers ou réguliers [1, 12]. L'image type est dite en « balle de coton » en rapport avec le refoulement tumoral du tissu avoisinant sans véritable envahissement [1]; cependant le caractère rassurant de l'imagerie contrastant avec l'âge avancé des patientes devrait faire craindre la malignité. Le carcinome colloïde mixte apparait sous forme de masse de contours irréguliers avec des limites mal définies, voire spéculées avec le tissu glandulaire [8, 13, 14]. Le nombre de spicules est inversement proportionnel à la quantité du mucus [8, 14, 15].

Les microcalcifications sont rares et sont généralement liées à la présence d'un carcinome in situ associé et apparaissent uniquement au niveau de la composante canalaire [1, 6]. La mammographie peut être normale dans 5 à 15% des cas [6]. Dans notre cas, la mammographie a mis en évidence des microcalcifications éparses et à l'étude histologique on note la présence d'une composante de carcinome canalaire.

L'échographie et la mammographie peuvent ainsi être d'une aide précieuse dans le diagnostic différentiel entre carcinome colloïde pur et mixte [16]. L'aspect échographique diffère selon le type de carcinome colloïde; le pur se traduit par une lésion lobulée, iso- ou hypoéchogène homogène de contours bien circonscrits, difficiles à différencier du tissu graisseux environnant, bien limitée avec renforcement postérieur, ce dernier étant expliqué par la quantité importante d'eau au sein de la tumeur et la transmission des ultrasons à travers le mucus. Le type mixte prend l'aspect d'une masse hypoéchgène hétérogène avec atténuation acoustique postérieure, ce qui reflète la nature infiltrative de la tumeur [6].

Il faut penser également au carcinome colloïde du sein à l'échographie devant une image complexe (liquide et solide) avec renforcement postérieure chez une patiente âgée [6, 17]. L'imagerie par résonance magnétique est de grand intérêt pour distinguer un carcinome colloïde pur d'un fibroadénome [1]. Macroscopiquement, on retrouve une masse tumorale, bien limitée, crépitant à la palpation, à surface gélatineuse, filant à la coupe. La consistance est molle, de couleur grisâtre ou gris jaune [6]. Histologiquement, on retrouve des ilots de cellules épithéliales régulières avec des lacs extensifs de mucus extracellulaires, séparés par des cloisons fibreuses grêles [6], les cellules tumorales sont petites avec un noyau sombre au sein duquel est visible un petit nucléole [4]. La distinction entre les types pur et mixte est capitale du fait de son impact pronostique:

- Le carcinome colloïde pur est caractérisé par la présence d'un tissu tumoral complètement entouré de mucus extracellulaire abondant, sans composante canalaire infiltrante. Ce tissu ne dépasse pas 10% du volume tumoral global [6]. La transition entre le mucus et le tissu conjonctif avoisinant est brusque; le mucus abondant joue le rôle de barrière mécanique, ce qui atténue l'invasion cellulaire et confère à ce type un caractère moins agressif par rapport au type mixte
- la transition entre mucus extracellulaire et tissu carcinomateux adjacent est progressive, les deux territoires se chevauchant par endroits.
- Les carcinomes colloïdes purs du sein sont dans la majorité des cas des carcinomes de bas grade (selon le score de SBR); l'association du carcinome colloïde pur à un carcinome intracanalaire a été rapportée dans 17% des cas [1].

Il est admis que les carcinomes colloïdes exposent moins à l'envahissement ganglionnaire que les autres types histologiques [8]. La fréquence des métastases ganglionnaires augmente en fonction de la taille tumorale [1]. Celle du carcinome mucineux pur est de l'ordre de 2 à 14% et de 45 à 64% dans les formes mixtes [8, 15]. Certains auteurs considèrent que la présence de métastases ganglionnaire est fortement liée à la présence d'une composante carcinomateuse non mucineuse associée qui pourrait être ignorée par un mauvais échantillonnage lors de l'examen macroscopique [16].

Certains auteurs suggèrent dans leur étude que le curage ganglionnaire axillaire ne devrait plus être systématique dans le carcinome mucineux pur du sein [8]. Mais d'autres ont démontré qu'un âge jeune était parmi les facteurs souvent associés à la présence d'adénopathie axillaires [8]. De ce fait, la technique du ganglion sentinelle serait néanmoins utile afin de détecter les patientes présentant des métastases ganglionnaires et de mieux adapter le traitement adjuvant [16]. Le curage ganglionnaire de notre patient a ramené 28 ganglions métastatiques sur 37 ganglions avec emboles vasculaires et rupture capsulaires.

L'étude immunohistochimique des récepteurs hormonaux pour l'‘strogène et la progestérone a souvent révélée une forte présence, plus particulièrement de l'‘strogène (91% des cas) [1, 7]. Les récepteurs hormonaux sont fortement positifs dans notre cas.

La prise en charge thérapeutique repose sur la chirurgie avec ou sans, chimiothérapie et hormonothérapie adjuvante. Un traitement chirurgical conservateur (tumorectomie) est préconisé pour les T1 et T2 suivie de radiothérapie. L'irradiation partielle et accélérée du sein est actuellement la plus recommandée après une chirurgie conservatrice. Une radiothérapie exclusive peut être tentée dans les formes inopérables pour des raisons locales ou générales [1]. Poortmans [1] a rapporté une réduction du risque de récidive locorégionale de 70% des patients traités par irradiation indépendamment de l'âge, des caractéristiques de la tumeur et de l'administration systémique de traitement. Ces constatations pourraient permettre de nuancer l'attitude thérapeutique chirurgicale systématique adoptée en général vis-à-vis des carcinomes colloïdes du sein. En clinique, il existe un effet complémentaire de la radiothérapie et du tamoxifène en situation adjuvante dans les cancers du sein opérés [1].

La chimiothérapie à base de doxorubicine combinée au paclitaxel semble avoir une bonne réponse dans les formes localement avancées de carcinome colloïde du sein [1, 9]. L'hormonothérapie est indiquée à chaque fois que les récepteurs hormonaux sont positifs [6, 8, 16].

La survie du carcinome colloïde est nettement supérieure aux autres types de cancers mammaires, notamment dans sa forme pure [6, 8, 15]. La survie à dix ans passe de 91% dans la forme pure à 46% dans la forme mixte [1] La survenue de métastases dans les carcinomes colloïdes purs est tardive [1]. L'envahissement ganglionnaire est le principal marqueur pronostique du carcinome colloïde du sein [6, 8]. Le risque relatif de rechute et de décès en cas d'envahissement ganglionnaire est de 2,69 [6, 8]. Le nombre élevé de ganglions envahis, ainsi que la rupture capsulaire sont des facteurs pronostiques péjoratifs. La quantité du mucus, la cellularité, la taille tumorale et la composante intracanailre sont des facteurs à impact pronostique incertain [8, 14, 15]. Il est certain que le pronostic des carcinomes colloïdes du sein est favorable, en particulier dans sa forme pure. Cependant, des rechutes tardives aussi bien locorégionales qu'à distance peuvent survenir, d'où l'intérêt d'une surveillance à long terme [1].

## Conclusion

Une masse d'allure bénigne en imagerie n'est pas toujours rassurante, surtout si elle survient chez une femme de plus de 60 ans. Puisqu'elle peut révéler un carcinome colloïde. La distinction entre la variante, pure et mixte qui est importante car l'attitude thérapeutique et l'incidence pronostique en dépendent. Le pronostic de la forme mixte qui rejoint celui des carcinomes canalaires infiltrants est moins bon que celui de la forme pure. Au total, il faut toujours évoquer un carcinome colloïde du sein à la mammographie devant une opacité circonscrite ou microlobulée chez une femme âgée; les limites bien circonscrites orientent à priori vers le type pur, alors que les marges mal définies orientent plus vers le type mixte[6, 17].
